# Supplementary material for: Long-term impact of PM2.5 on mortality is exacerbated when wildfire events occur
Source: ArXiv. 2025 May 22:arXiv:2505.16613v1. Preprint. [Version 1] (PMC12136479)
Supplement: Supplement 1 [file NIHPP2505.16613v1-supplement-1.pdf]

Supplementary figures and tables

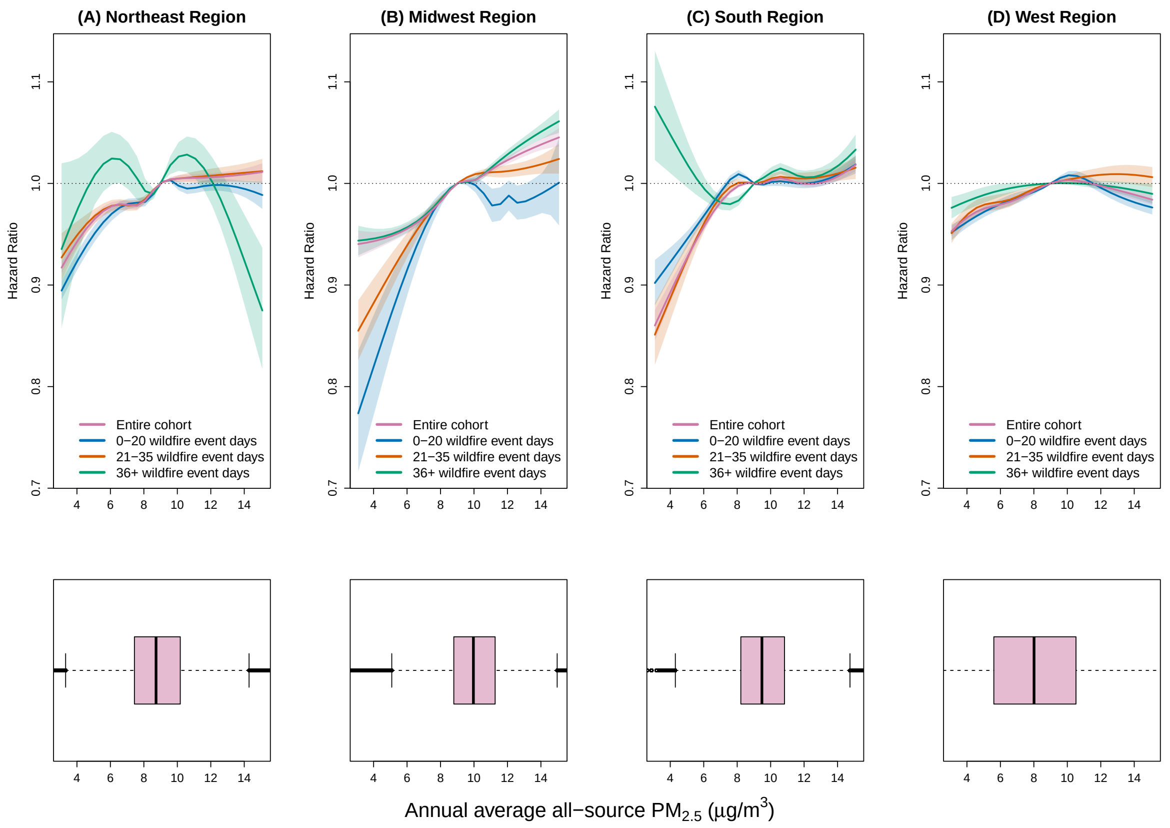

Figure S1: **Mortality hazard ratio with 95% confidence intervals and all-source $PM_{2.5}$ distribution stratifying by region.** The plots illustrate the exposure-response curve and distribution of all-source $PM_{2.5}$ values stratifying by region, ranging from the 1st to the 99st percentile of the entire cohort all-source $PM_{2.5}$ distribution. The top plots represent the hazard ratio of mortality compared to the current NAAQS for annual all-source $PM_{2.5}$($9\ \mu g/m^3$) stratifying by region. The lower plots represent the distribution of all-source $PM_{2.5}$ for each region.

Variables	Medicare beneficiaries
Number of individuals	60,999,431
Number of deaths	17,608,624 (28.9%)
Total person years	391,702,092
Median years of follow-up	9
Individual-level characteristics	
Age at entry (years)	
65–74 (%)	88.9
75–84 (%)	9.4
85–94 (%)	1.5
95 or above (%)	0.2
Sex	
Female (%)	55.4
Race and ethnicity	
Hispanic (%)	2.1
Native American and Alaska Native (%)	0.3
Non-Hispanic Asian (%)	2.1
Non-Hispanic Black (%)	9.0
Non-Hispanic White (%)	83.4
Medicaid eligibility	
Eligible (%)	12.9
Region of residence	
Northeast (%)	20.15
Midwest (%)	22.15
West (%)	20.67
South (%)	37.03
Area-level risk factors	
Ever smoked (%)	46.7
Below poverty level (%)	10.1
Less than high school education (%)	23.5
Owner-occupied housing (%)	71.6
Hispanic (%)	9.5
Non-Hispanic Black (%)	8.9
Population density (persons/km^2)	1556.0 (5160.5)
Mean BMI (kg/m^2)	27.80 (1.02)
Median household income ($1000)	53.1 (23.0)
Median home value ($1000)	187.7 (157.8)
Meteorological variables, mean (SD)	
Summer temperature (°C)	29.7 (3.8)
Winter temperature (°C)	7.4 (7.3)
Summer relative humidity (%)	86.8 (11.8)
Winter relative humidity (%)	85.6 (7.2)
All-source $PM_{2.5}$ concentration ($\mu g/m^3$)	9.0 (2.7)
Wildfire $PM_{2.5}$ concentration ($\mu g/m^3$)	0.4 (0.3)
Non-zero wildfire $PM_{2.5}$ days per year	31.8 (21.7)

Table S1: **Characteristics for the Medicare study cohort, 2006–2016.** Mortality and individual-level characteristics were obtained from the Centers for Medicare and Medicaid Services (CMS), and ZIP code socioeconomic status (SES) was obtained from the 2000 and 2010 Census and the 2005–2012 American Community Surveys (ACS) and county-level behavioral risk factor variables were obtained from the Centers for Disease Control and Prevention. All-source $PM_{2.5}$ and wildfire $PM_{2.5}$ concentrations were obtained respectively from [3] and [25]. Meteorological variables were obtained from Gridmet via Google Earth Engine.

**Stratification**	**Main**	**Poverty**		**Region**			
		>15%	≤15%	Northeast	Midwest	South	West
Entire cohort	5	6	5	6	6	6	6
0–20 days	6	6	6	6	6	6	6
21–35 days	4	5	4	5	3	5	6
36+ days	6	5	6	5	5	6	3

Table S2: **Optimal degrees of freedom for spline terms.** Degrees of freedom selected for the natural spline of annual average all-source $PM_{2.5}$ in each wildfire, poverty and region category stratum. Values were selected to minimize the Akaike Information Criterion (AIC).
